# *In Vitro* Antifungal Activity of Ibrexafungerp (SCY-078) Against Contemporary Blood Isolates From Medically Relevant Species of *Candida*: A European Study

**DOI:** 10.3389/fcimb.2022.906563

**Published:** 2022-05-16

**Authors:** Guillermo Quindós, Katherine Miranda-Cadena, Rosario San-Millán, Katyna Borroto-Esoda, Emilia Cantón, María José Linares-Sicilia, Axel Hamprecht, Isabel Montesinos, Anna Maria Tortorano, Anna Prigitano, Matxalen Vidal-García, Cristina Marcos-Arias, Andrea Guridi, Ferran Sanchez-Reus, Jesús Machuca-Bárcena, Manuel Antonio Rodríguez-Iglesias, Estrella Martín-Mazuelos, Carmen Castro-Méndez, Leyre López-Soria, Alba Ruiz-Gaitán, Marcelo Fernandez-Rivero, Damaris Lorenzo, Javier Capilla, Antonio Rezusta, Javier Pemán, Josep Guarro, Joana Pereira, Célia Pais, Orazio Romeo, Guillermo Ezpeleta, Nerea Jauregizar, David Angulo, Elena Eraso

**Affiliations:** ^1^ Laboratorio de Micología Médica, UFI 11/25, Departamento de Inmunología, Microbiología y Parasitología, Facultad de Medicina y Enfermería, Universidad del País Vasco/Euskal Herriko Unibertsitatea (UPV/EHU), Bilbao, Spain; ^2^ SCYNEXIS, Inc., Jersey City, NJ, United States; ^3^ Instituto de Investigación Sanitaria, Hospital Universitario y Politécnico La Fe, Valencia, Spain; ^4^ Research Group GC24, Maimonides Biomedical Research Institute of Cordoba (IMIBIC), Department of Microbiology, Facultad de Medicina y Enfermería, Universidad de Córdoba, Córdoba, Spain; ^5^ University Hospital Cologne, Cologne and Institute for Medical Microbiology and Virology, University of Oldenburg, Oldenburg, Germany; ^6^ Microbiology Department, LHUB-ULB, Hôpital Erasme, Brussels, Belgium; ^7^ Dipartimento di Scienze Biomediche per la Salute, Università degli Studi di Milano, Milan, Italy; ^8^ Servicio de Microbiología, Hospital Universitario Miguel Servet, Zaragoza, Spain; ^9^ Servei de Microbiologia, Hospital de la Santa Creu i Sant Pau, Barcelona, Spain; ^10^ Área de Microbiología, Departamento de Biomedicina, Biotecnología y Salud Pública, Facultad de Medicina, Universidad de Cádiz, Cádiz, Spain; ^11^ Servicio de Microbiología, Hospital Universitario de Valme, Sevilla, Spain; ^12^ Servicio de Microbiología, Hospital Universitario de Cruces and BioCruces Bizkaia, Barakaldo, Spain; ^13^ Microbiology Unit, Medical School, Universitat Rovira i Virgili, Reus, Spain; ^14^ Centre of Molecular and Environmental Biology (CBMA), Department of Biology, University of Minho, Braga, Portugal; ^15^ Department of Chemical, Biological, Pharmaceutical and Environmental Sciences, University of Messina, Messina, Italy; ^16^ Servicio de Microbiología, Complejo Hospitalario de Navarra, Pamplona and Departamento de Medicina Preventiva y Salud Pública, Facultad de Medicina y Enfermería, Universidad del País Vasco/Euskal Herriko Unibertsitatea (UPV/EHU), Bilbao, Spain; ^17^ Departamento de Farmacología, Facultad de Medicina y Enfermería, Universidad del País Vasco/Euskal Herriko Unibertsitatea (UPV/EHU), Bilbao, Spain

**Keywords:** antifungal testing, antifungal resistance, *Candida*, ibrexafungerp, SCY-078, EUCAST, caspofungin, micafungin

## Abstract

**Background:**

Ibrexafungerp (SCY-078) is the newest oral and intravenous antifungal drug with broad activity, currently undergoing clinical trials for invasive candidiasis.

**Objective:**

The aim of this study was to assess the *in vitro* activity of ibrexafungerp and comparators against a collection of 434 European blood isolates of *Candida*.

**Methods:**

Ibrexafungerp, caspofungin, fluconazole, and micafungin minimum inhibitory concentrations (MICs) were collected from 12 European laboratories for 434 blood isolates, including 163 *Candida albicans*, 108 *Candida parapsilosis*, 60 *Candida glabrata*, 40 *Candida tropicalis*, 29 *Candida krusei*, 20 *Candida orthopsilosis*, 6 *Candida guilliermondii*, 2 *Candida famata*, 2 *Candida lusitaniae*, and 1 isolate each of *Candida bracarensis, Candida catenulata*, *Candida dubliniensis*, and *Candida kefyr*. MICs were determined by the EUCAST broth microdilution method, and isolates were classified according to recommended clinical breakpoints and epidemiological cutoffs. Additionally, 22 *Candida auris* from different clinical specimens were evaluated.

**Results:**

Ibrexafungerp MICs ranged from 0.016 to ≥8 mg/L. The lowest ibrexafungerp MICs were observed for *C. albicans* (geometric MIC 0.062 mg/L, MIC range 0.016–0.5 mg/L) and the highest ibrexafungerp MICs were observed for *C. tropicalis* (geometric MIC 0.517 mg/L, MIC range 0.06–≥8 mg/L). Modal MICs/MIC_50_s (mg/L) against *Candida* spp. were 0.125/0.06 for *C. albicans*, 0.5/0.5 for *C. parapsilosis*, 0.25/0.25 for *C. glabrata*, 0.5/0.5 for *C. tropicalis*, 1/1 for *C. krusei*, 4/2 for *C. orthopsilosis*, and 0.5/0.5 for *C. auris*. Ibrexafungerp showed activity against fluconazole- and echinocandin-resistant isolates. If adopting wild-type upper limits, a non-wild-type phenotype for ibrexafungerp was only observed for 16/434 (3.7%) isolates: 11 (4.6%) *C. parapsilosis*, 4 (5%) *C. glabrata*, and 1 (2.5%) *C. tropicalis*.

**Conclusion:**

Ibrexafungerp showed a potent *in vitro* activity against *Candida*.

## Introduction

Invasive candidiasis (IC) is the most common healthcare-associated invasive mycosis, being a major cause of human morbidity and mortality. *Candida albicans* is the most prevalent etiology, but other species, such as *Candida glabrata*, *Candida parapsilosis*, *Candida krusei* (*Pichia kudriavzevii*), and, more recently, *Candida auris*, are increasing causes of IC. These emergent species are usually less susceptible to current antifungal drugs. Although *Candida* isolates displaying antifungal resistance are still uncommon, they are increasingly reported worldwide. Therapy of IC is an unsolved clinical challenge and, for this reason, monitoring antifungal susceptibility patterns and resistance mechanisms is of utmost importance. Moreover, new antifungal drugs are needed as the number of available antifungal drug classes, and particularly those for oral administration, is limited (Arendrup and Patterson, 2017; Quindós et al., 2018; Fuller et al., 2019; Pfaller et al., 2019).

Ibrexafungerp (formerly SCY-078) is a semisynthetic triterpenoid glycoside derived from enfumafungin, which is structurally different from echinocandins and form a new class of antifungal drugs called “fungerps” that strongly inhibit fungal 1,3-β-glucan synthase (Davis et al., 2020). Even ibrexafungerp and echinocandins share similar mechanisms of action, and their binding sites to the target enzyme is not the same, resulting in very limited cross-resistance (Jiménez-Ortigosa et al., 2017; Pfaller et al., 2017). Ibrexafungerp displays significant *in vitro* and *in vivo* activities against azole- and echinocandin-resistant isolates of *Candida* species, including biofilm-forming strains (Jiménez-Ortigosa et al., 2014; Pfaller et al., 2017; Schell et al., 2017; Gamal et al., 2021).

Ibrexafungerp aims to be the first orally and intravenously available glucan synthase inhibitor useful in the treatment of life-threatening fungal infections (Davis et al., 2020) as well as superficial ones, such as vulvovaginal candidiasis (Schwebke et al., 2021; Sobel et al., 2022). Currently, there are 13 listed clinical trials for ibrexafungerp, eight of which have been completed (https://ClinicalTrials.gov/; accessed on March 8, 2022).

In the current study, we have determined the anti-*Candida in vitro* activity of ibrexafungerp, caspofungin, fluconazole, and micafungin against 434 European *Candida* blood isolates analyzed in 12 European laboratories.

## Materials and Methods

### Microorganisms

*In vitro* susceptibility of a collection of 434 *Candida* blood isolates (2016–2018) from 434 patients was determined at 12 laboratories from Belgium, Germany, Italy, Portugal, and Spain. Each laboratory studied its own clinical isolates. The collection included 163 C*. albicans*, 108 C*. parapsilosis*, 60 C*. glabrata*, 40 C*. tropicalis*, 29 C*. krusei*, 20 *Candida orthopsilosis*, 6 *Candida guilliermondii* (*Meyerozyma guilliermondii*), 2 *Candida famata* (*Debaryomyces hansenii*), 2 *Candida lusitaniae* (*Clavispora lusitaniae*), and 1 isolate each of *Candida bracarensis, Candida catenulata* (*Diutina catenulata*), *Candida dubliniensis*, and *Candida kefyr* (*Kluyveromyces marxianus*). Additionally, 22 C*. auris* from different clinical specimens were evaluated: Eight isolates were from blood, seven from oral specimens, and 7 from urine (Ruiz-Gaitán et al., 2017; Ruiz-Gaitán et al., 2018). Isolates were identified by phenotypic methods, MALDI-TOF (proteomic method), and, when needed, internal transcribed spacer (ITS) sequencing (genotypic method) (Miranda-Zapico et al., 2011). *C. parapsilosis* ATCC 22019 and *C. krusei* ATCC 6258 were included as quality control (QC) strains. Reference strain *C. albicans* ATCC 64550 was also included as recommended by EUCAST for detecting variation in echinocandin activities. Prior to testing, each isolate was cultured onto Sabouraud dextrose agar and/or CHROMagar Candida medium (Becton Dickinson, Sparks, MD, USA) to ensure purity and viability. QC and reference strain were included every testing day and all their results were within published ranges.

### Antifungal Drugs and Susceptibility Testing

All isolates were tested for *in vitro* susceptibility to ibrexafungerp, caspofungin, fluconazole, and micafungin using the EUCAST broth microdilution method (Arendrup et al., 2020a; Arendrup et al., 2020b; EUCAST, 2020; EUCAST, 2021). The reference powder of ibrexafungerp was obtained from the manufacturer (SCYNEXIS Inc., Jersey City, NJ, USA). Stock solutions were prepared in dimethyl sulfoxide (DMSO), and the final range of ibrexafungerp, caspofungin, and micafungin concentrations tested was 0.008 to 8 mg/L except for fluconazole, which ranged from 0.125 to 128 mg/L. The EUCAST method was performed by using RPMI 1640 with 2% glucose and buffered to pH 7.0 with 0.165 M morpholinopropane sulphate (MOPS). Panels were inoculated with a final standardized cell concentration of 0.5–2.5 × 10^5^ cells/ml. Panels were read spectrophotometrically at 450 nm after 24 h of incubation at 36 ± 1°C. Although caspofungin presents excessive inter-laboratory variation in minimum inhibitory concentration (MIC) results by the EUCAST broth microdilution method (Espinel-Ingroff et al., 2013), MICs were calculated for comparison purposes.

### Data Analysis

MIC values were determined as the lowest concentration of antifungal drug that inhibited 50% of growth compared to that of the growth control. For individual species of *Candida* for which ten or more isolates were tested, summary descriptive statistics, including MIC ranges, modal MIC (MM) (the most reported MIC), geometric mean MIC (GM), MIC at which 50% of the isolates was inhibited (MIC_50_), and MIC at which 90% of the isolates was inhibited (MIC_90_), were calculated for each species and drug. High off-scale MIC results were converted to the next highest concentration, and low off-scale MIC results were left unchanged. Epidemiological cutoff values (ECVs) were used to differentiate wild-type (WT) (without acquired resistance mechanisms) from non-wild-type (NWT) (may harbor acquired resistance mechanisms) isolates. For antifungal drugs, such as ibrexafungerp and caspofungin, or/and species, such as *C. auris* and *C. orthopsilosis*, without EUCAST ECVs, WT upper limits (WTUL) were used as the susceptibility cutoff values and MICs > 2 dilution steps above the MM were regarded as NWT (**Table 1**). Comparison of efficacy among antifungal drugs was based on MIC_90_ differences: Those antifungal drugs with a difference of one double dilution were considered to have a similar activity (Wiederhold, 2021). This comparison should not be considered absolute, as other pharmacokinetics and pharmacodynamics (PK/PD) parameters should be taken into account. The study was approved by the Ethics Committee of the Universidad del País Vasco/Euskal Herriko Unibertsitatea (UPV/EHU, Bilbao, Spain, CEIAB Ethics reference number M30_2015_248).

**Table 1:** EUCAST clinical breakpoints (CBPs) and epidemiological cutoff values (ECVs).

Species	Antifungal drug	CBPs (mg/L)		ECVs (mg/L)	
		Susceptible	Resistant	Wild-type	Non Wild-type
*Candida albicans*					
(*n* = 163)	Ibrexafungerp	–	–	≤0.5	>0.5
	Caspofungin	–	–	≤0.5	>0.5
	Micafungin	≤0.016	>0.016	≤0.016	>0.016
	Fluconazole	≤2	>4	≤0.5	>0.5
*Candida auris*					
(*n* = 22)	Ibrexafungerp	–	–	≤2	>2
	Caspofungin	–	–	≤1	>1
	Micafungin	–	–	≤0.5	>0.5
	Fluconazole	≤2	>4	≤64	>64
*Candida glabrata*					
(*n* = 60)	Ibrexafungerp	–	–	≤1	>1
	Caspofungin	–	–	≤1	>1
	Micafungin	≤0.03	>0.03	≤0.03	>0.03
	Fluconazole	≤0.001	>16	≤16	>16
*Candida krusei*					
(*n* = 29)	Ibrexafungerp	–	–	≤4	>4
	Caspofungin	–	–	≤2	>2
	Micafungin	–	–	≤0.25	>0.25
	Fluconazole	–	–	≤128	>128
*Candida parapsilosis*					
(*n* = 108)	Ibrexafungerp	–	–	≤2	>2
	Caspofungin	–	–	≤8	>8
	Micafungin	≤2	>2	≤2	>2
	Fluconazole	≤2	>4	≤2	>2
*Candida orthopsilosis*					
(*n* = 20)	Ibrexafungerp	–	–	≤8	>8
	Caspofungin	–	–	≤8	>8
	Micafungin	–	–	≤4	>4
	Fluconazole	≤2	>4	≤2	>2
*Candida tropicalis*					
(*n* = 40)	Ibrexafungerp	–	–	≤2	>2
	Caspofungin	–	–	≤1	>1
	Micafungin	–	–	≤0.06	>0.06
	Fluconazole	≤2	>4	≤1	>1

Gray shaded area: Potentially non-wild-type MICs > 2 dilution steps above the modal MIC.

## Results

MICs of ibrexafungerp against all species were tested *in vitro* ranging from 0.016 to ≥8 mg/L (MIC_90_ 2 mg/L). These values were comparable to those of caspofungin and micafungin (MIC_90_ 2 mg/L). The lowest ibrexafungerp MICs were observed for *C. albicans* (GM 0.062 mg/L, MIC range 0.016–0.5 mg/L, MIC_90_ 0.125 mg/L) and the highest ibrexafungerp MICs were observed for *C. tropicalis* (GM 0.517 mg/L, MIC range 0.06–≥8 mg/L, MIC_90_ 2 mg/L) (**Table 2**). Echinocandins MIC values for *C. albicans* ranged from ≤ 0.008 mg/L to 2 mg/L for micafungin (MM 0.008 mg/L, GM 0.001 mg/L, MIC_90_ 0.016 mg/L) and from 0.016 mg/L to 2 mg/L for caspofungin (MM 0.125 mg/L, GM 0.104 mg/L, MIC_90_ 0.125 mg/L). For the three isolates with elevated caspofungin or/and micafungin MICs, ibrexafungerp MIC range was 0.06 mg/L to 0.25 mg/L. In the current report, ibrexafungerp MICs ranged from 0.016 mg/L to 8 mg/L for 108 C*. parapsilosis* isolates (MM 0.5 mg/L, GM 0.660 mg/L, MIC_90_ 4 mg/L) (**Table 2**). If we consider ibrexafungerp WTULs (>2 mg/L) obtained in this study, 11 C*. parapsilosis* isolates were NWT (4.6%). These NWT isolates were inhibited by ≤1 mg/L of fluconazole and by ≤4 mg/L of caspofungin or micafungin. We also observed that for six *C. parapsilosis* NWT (5.6%) and two resistant isolates to fluconazole (0.9%), the MIC range of ibrexafungerp was 0.5–2 mg/L. According to MIC90s, ibrexafungerp showed comparable values with caspofungin (MM 1 mg/L, GM 0.846 mg/L, MIC_90_ 2 mg/L) and micafungin (MM 2 mg/L, GM 0.933 mg/L, MIC_90_ 2 mg/L). Moreover, two isolates resistant to micafungin were inhibited by 2 mg/L of ibrexafungerp.

**Table 2:** Summary of ibrexafungerp and comparators’ *in vitro* antifungal activities against blood isolates from medically relevant species of *Candida*.

Species/Antifungal drugs		No. of isolates at MIC (mg/L) (Cumulative %)																			Total	MIC (mg/L)					
		≤0.008	0.016		0.03	0.06	0.125		0.25	0.5	1	2	4	8		16		32	64	≥128		Range	Mode	GM	50		90
*Candida albicans* (*n* = 163)																											
Ibrexafungerp		0		7 (4.3)	50 (35)	48 (64.4)	50 (95.1)	6 (98.8)		2 (100)	0	0	0	0		0		–	–	–	163	0.016-0.5	0.125	0.062	0.06		0.125
Caspofungin		0		3 (1.9)	7 (6.1)	38 (29.4)	100 (90.8)	8 (95.7)		4 (98.2)	1 (98.7)	1 (100)	0	0		0		–	–	–	163	0.016-2	0.125	0.104	0.125		0.125
Micafungin		82 (50.3)		79 (98.8)	**1 (99.4)**	**0**	**0**	**0**		**0**	**0**	**1 (100)**	**0**	**0**		**0**		–	–	–	163	≤0.008–2	0.008	0.001	0.008		0.016
Fluconazole		–		–	–	–	56 (34.4)	90 (89.6)		14 (98.2)	1 (98.8)	0	0	**0**		**1 (99.4)**		**0**	**0**	**1 (100)**	163	0.125-≥128	0.25	0.226	0.25		0.5
*Candida parapsilosis* (*n* = 108)																											
Ibrexafungerp		0		6 (5.6)	0	1 (6.5)	3 (9.3)	10 (18.5)		38 (53.7)	24 (75.9)	15 (89.8)	6 (95.4)	5 (100)		0		–	–	–	108	0.016-8	0.5	0.660	0.5	4	
Caspofungin		0		0	0	1 (0.9)	5 (5.6)	3 (8.3)		26 (32.4)	51 (79.6)	19 (97.2)	3 (100)	0		0		–	–	–	108	0.06–4	1	0.846	1	2	
Micafungin		3 (3.3)		2 (4.6)	1 (5.6)	0	0	5 (10.2)		15 (24.1)	32 (53.7)	48 (98.1)	**2 (100)**	**0**		**0**		–	–	–	108	≤0.008–4	2	0.933	1	2	
Fluconazole		–		–	–	–	0	7 (6.5)		68 (69.4)	21 (88.9)	6 (94.4)	4 (98.1)	**0**		**0**		**1 (99.1)**	**0**	**1 (100)**	108	0.25-≥128	0.5	0.698	0.5	2	
*Candida glabrata* (*n* = 60)																											
Ibrexafungerp	0			2 (3.3)	0	1 (5)	10 (21.7)	22 (58.3)		16 (85)	5 (93.3)	1 (95)	2 (98.3)	1 (100)		0		–	–	–	60	0.016-8	0.25	0.322	0.25	1	
Caspofungin	0			0	0	3 (5)	17 (33.3)	19 (65)		15 (90)	4 (96.7)	0	1 (98.3)	0		1 (100)		–	–	–	60	0.06->8	0.25	0.280	0.25	0.5	
Micafungin	18 (30)			30 (80)	3 (85)	**1 (86.7)**	**1 (86.7)**	**0**		**1 (90)**	**4 (96.7)**	**2 (100)**	**0**	**0**		**0**		–	–	–	60	≤0.008-2	0.016	0.023	0.016	0.5	
Fluconazole	–			–	–	–	0	2 (3.3)		4 (10)	1 (11.7)	11 (30)	6 (40)	12 (60)		5 (68.3)		**2 (71.7)**	**6 (81.7)**	**11 (100)**	60	0.25-≥128	8	9.514	8	≥128	
*Candida tropicalis* (*n* = 40)																											
Ibrexafungerp	0			0	0	2 (5)	3 (12.5)	9 (35)		12 (65)	8 (85)	5 (97.5)	0	0	1 (100)		–		–	–	40	0.06->8	0.5	0.517	0.5	2	
Caspofungin	0			0	0	1 (2.5)	15 (40)	19 (87.5)		4 (97.5)	0	0	0	0	1 (100)		–		–	–	40	0.06->8	0.25	0.221	0.25	0.5	
Micafungin	1 (2.5)			7 (20)	24 (80)	7 (97.5)	0	0		0	1 (100)	0	0	0	0		–		–	–	40	≤0.008-1	0.03	0.032	0.03	0.06	
Fluconazole	–			–	–	–	1 (2.5)	8 (22.5)		9 (45)	6 (60)	1 (62.5)	1 (65)	**0**	**0**		**2 (70)**		**12 (100)**	**0**	40	0.125-64	64	3.364	2	64	
*Candida krusei* (*n* = 29)																											
Ibrexafungerp	0			0	0	0	1 (3.4)	2 (10.3)		10 (44.8)	16 100)	0	0	0	0		–		–	–	29	0.125-1	1	0.666	1	1	
Caspofungin	0			0	0	0	0	7 (24.1)		18 (86.2)	4 (100)	0	0	0	0		–		–	–	29	0.25 – 1	0.5	0.465	0.5	1	
Micafungin	0			0	1 (3.4)	1 (6.9)	25 (93.1)	2 (100)		0	0	0	0	0	0		–		–	–	29	0.03-0.25	0.125	0.122	0.125	0.125	
Fluconazole	–			–	–	–	0	0		0	0	0	0	0	4 (13.8)		18 (75.9)		5 (93.1)	2 (100)	29	16-≥128	32	37.828	32	64	
*Candida orthopsilosis* (*n* = 20)																											
Ibrexafungerp	0			0	0	1 (5)	1 (10)	0		3 (25)	2 (35)	3 (50)	10 (100)	0	0		–		–	–	20	0.06-4	4	1.566	2	4	
Caspofungin	0			0	0	0	0	1 (5)		6 (35)	1 (40)	8 (80)	4 (100)	0	0		–		–	–	20	0.25-4	2	1.320	2	4	
Micafungin	0			0	0	1 (5)	0	1 (10)		5 (35)	10 (85)	3 (100)	0	0	0		–		–	–	20	0.06-2	1	0.756	1	1	
Fluconazole	–			–	–	–	1 (5)	0		12 (65)	3 (80)	2 (90)	1 (95)	**0**	**1 (100)**		**0**		**0**	**0**	20	0.125-16	0.5	0.783	0.5	2	

Resistant isolates according to EUCAST criteria for fluconazole and micafungin are highlighted in bold type. Accepted and potentially non-wild-type MIC ranges are shaded for blood isolates with MICs > 2 dilution steps above the modal MIC.

Ibrexafungerp also displayed potent *in vitro* activity against 60 C*. glabrata* isolates (MIC range 0.016–8 mg/L, MM 0.25 mg/L, GM 0.322 mg/L, MIC_90_ 1 mg/L) (**Table 2**). Sixteen *C. glabrata* isolates resistant to fluconazole were inhibited by ≤1 mg/L of ibrexafungerp. Among 9 C*. glabrata* isolates with elevated MICs to caspofungin or/and micafungin, the MIC range for ibrexafungerp was 0.25 mg/L to 4 mg/L with a MIC_50_ of 1 mg/L. It was notable that whereas the increase in MM between *C. glabrata* WT and NWT isolates was 4-fold for caspofungin and 63-fold for micafungin, MM values for ibrexafungerp did not increase (**Table 3**).

**Table 3:** MICs distribution of ibrexafungerp and comparator antifungal drugs against echinocandin wild-type (WT) and non-wild-type/resistant (NWTR) *Candida* spp. Isolates.

Species	Phenotype (no. of isolates)	Modal MIC (MIC range) [mg/L]			
		Ibrexafungerp	Caspofungin	Micafungin	Fluconazole
*Candida albicans*	WT (160)	0.125 (0.016–2)	0.125 (0.016–0.5)	0.016 (≤0.008–0.016)	0.25 (0.125–≥128)
	NWTR (3)	− (0.03–0.25)	− (0.125–2)	− (0.03–>8)	0.25 (0.25)
*Candida parapsilosis*	WT (106)	0.5 (0.016–8)	1 (0.06–4)	2 (≤0.008–2)	0.25 (0.25–≥128)
	NWTR (2)	2 (2)	− (1–2)	4 (4)	− (0.25–4)
*Candida glabrata*	WT (51)	0.25 (0.016–8)	0.25 (0.06–0.5)	0.016 (≤0.008–0.03)	8 (0.25–≥128)
	NWTR (9)	0.25 (0.25–4)	1 (0.5–>8)	1 (0.06–2)	128 (0.5–128)
*Candida tropicalis*	WT (39)	0.25 (0.03–>8)	0.25 (0.03–>8)	0.5 (0.03–>8)	0.25 (0.03–16)
	NWTR (1)	>8 (>8)	>8 (>8)	0.5 (0.5)	0.25 (0.25)
*Candida orthopsilosis*	WT (16)	4 (0.06–4)	2 (0.25–2)	1 (0.06–2)	0.5 (0.125–16)
	NWTR (4)	4 (2–4)	4 (4)	1 (1–2)	0.5 (0.5–1)
*Candida auris*	WT (16)	0.5 (0.5–4)	0.25 (0.25–2)	0.25 (0.25–1)	≥128 (≥128)
	NWTR (6)	0.5 (0.5–8)	>8 (2–>8)	1 (1–>8)	≥128 (≥128)
*Candida guilliermondii*	WT (5)	2 (2–4)	0.5 (0.5–1)	0.5 (0.5–1)	0.5 (0.5–64)
	NWTR (1)	4 (4)	8 (8)	0.008 (0.008)	8 (8)

For antifungals without established epidemiological cutoff points, the points established in **Table 1** (shaded area) have been taken into account.

*In vitro* activity of ibrexafungerp was also observed against 40 C*. tropicalis* blood isolates and MICs ranged from 0.06 mg/L to ≥8 mg/L (MM 0.5 mg/L, GM 0.517 mg/L, MIC_90_ 2 mg/L) (**Table 2**). Against 14 C*. tropicalis* resistant to fluconazole, ibrexafungerp MIC range was 0.25–2 mg/L (MIC_90_ 1 mg/L).

Moreover, ibrexafungerp showed high *in vitro* activity against 29 C*. krusei* (MIC range 0.125–1 mg/L, MM 1 mg/L, GM 0.666 mg/L, MIC_90_ were 1 mg/L). MIC values of micafungin ranged from 0.03 mg/L to 0.25 mg/L (MM 0.125 mg/L, GM 0.122 mg/L, MIC_90_ 0.125 mg/L) and from 0.25 mg/L to 1 mg/L for caspofungin (MM 0.5 mg/L, GM 0.465 mg/L, MIC_90_ 1 mg/L). Additionally, all *C. auris* isolates were resistant *in vitro* to fluconazole (MIC ≥ 128 mg/L) while ibrexafungerp showed activity (MIC range 0.5 mg/L to 8 mg/L, MM 0.5 mg/L, GM 0.753 mg/L, MIC_90_ 2 mg/L) (**Table 4**). *C. auris* urinary isolates showed higher MICs (data not shown). Against this species, the activity of ibrexafungerp was similar to the activity of micafungin (MIC range 0.125–>8 mg/L, MM 0.125 mg/L, GM 0.377 mg/L, MIC_90_ 4 mg/L) and 8-fold more active than caspofungin (MIC range 0.25–>8 mg/L, MM 0.25 mg/L, GM 0.585 mg/L, MIC_90_ >8 mg/L). Among six isolates with elevated caspofungin or/and micafungin MICs, ibrexafungerp MICs ranged from 0.5 mg/L to 8 mg/L (MIC_50_ 0.5 mg/L).

**Table 4:** Summary of ibrexafungerp and comparators’ *in vitro* antifungal activities against 22 clinical isolates of *Candida auris*.

Antifungal drugs	No. of blood/any origin isolates at MIC (mg/L) (Cumulative %)															Total	MIC				
	≤0.008	0.016	0.03	0.06	0.125	0.25	0.5	1	2	4	8	16	32	64	≥128		Range	Mode	GM	50	90
Ibrexafungerp	0	0	0	0	0	0	5/16 (62.5/72.7)	½ (75/81.8)	½ (87.5/90.9)	0/1 (87.5/95.5)	1/1 (100)	0	–	–	–	8/22	0.5-8	0.5	0.753	0.5	2
Caspofungin	0	0	0	0	0	7/16 (87.5/72.7)	0	0	0/1 (87.5/77.3)	0	0	1/5 (100)	–	–	–	8/22	0.25->8	0.25	0.585	0.25	>8
Micafungin	0	0	0	0	6/13 (75/59.1)	0/1 (75/63.6)	0	¼ (87.5/81.8)	0/1 (87.5/86.4)	0/1 (87.5/90.9)	0/1 (87.5/95.5)	1/1 (100)	–	–	–	8/22	0.125->8	0.125	0.377	0.125	4
Fluconazole	0	0	0	0	0	0	0	0	0	0	0	0	0	0	8/22 (100)	8/22	≥128	≥128	211.905	≥128	≥128

Potentially non-wild-type MIC ranges are shaded for blood isolates with MICs > 2 dilution steps above the modal MIC.

MICs of ibrexafungerp were ≤ 1 mg/L for the 2 C*. famata* isolates and 1 isolate each of *C. bracarensis*, *C. catenulata*, *C. dubliniensis*, and *C. kefyr* (**Table 5**). Ibrexafungerp MIC_50_ was 2 mg/L for the 6 C*. guilliermondii* blood isolates with a MIC range from 2 to 4 mg/L, being twofold less active than caspofungin or micafungin.

**Table 5:** Activities of ibrexafungerp and comparator antifungal drugs against other species of *Candida* blood isolates.

Species	Isolate reference	MIC (mg/L)			
		Ibrexafungerp	Caspofungin	Micafungin	Fluconazole
*Candida bracarensis*	18-3133Br	0.25	0.5	0.016	4
*Candida catenulata*	17-011	0.5	0.5	2	1
*Candida dubliniensis*	18-10677-2C	0.5	0.25	0.008	0.25
*Candida famata*	17-012	0.5	1	2	0.5
*Candida famata*	10-10647-1C	1	1	0.125	1
*Candida guilliermondii*	10-606-1C	2	1	1	1
*Candida guilliermondii*	17-23377-2C	2	0.5	0.5	0.5
*Candida guilliermondii*	17-25047C	2	0.5	0.5	0.5
*Candida guilliermondii*	17-341	2	1	1	64
*Candida guilliermondii*	17-014	4	0.5	0.5	0.5
*Candida guilliermondii*	17-324	4	8	0.008	8
*Candida kefyr*	18-001	0.25	0.5	0.125	0.25
*Candida lusitaniae*	17-018	8	0.5	0.125	0.125
*Candida lusitaniae*	11-423-1C	4	0.5	0.06	32

## Discussion

The present study aimed to evaluate the *in vitro* activity of ibrexafungerp against a collection of 434 European blood isolates of *Candida*. Ibrexafungerp showed a potent *in vitro* activity against *Candida*, with MICs ranging from 0.016 to 16 mg/L. The lowest ibrexafungerp MICs were observed against *C. albicans* and the highest ibrexafungerp MICs were observed against *C. tropicalis* Moreover, ibrexafungerp also displayed remarkable activity against fluconazole- and echinocandin-resistant isolates.

Five species of *Candida* (*C. albicans*, *C. parapsilosis*, *C. glabrata*, *C. tropicalis*, and *C. krusei*) cause more than 90% of IC (Quindós, 2014; Arendrup and Patterson, 2017; Quindós et al., 2018). *C. albicans* remains the predominant cause, but there is an evident shift in the etiology, and IC caused by other species less susceptible to current antifungal drugs is becoming more frequent (Fuller et al., 2019; Pfaller et al., 2019). Echinocandins, such as anidulafungin, caspofungin, and micafungin, are considered first-line therapy for IC because they possess fungicidal activity against *Candida* (Gil-Alonso et al., 2015a; Gil-Alonso et al., 2015b). However, emergence of azole- and echinocandin-resistant [multidrug-resistant (MDR)] isolates has been reported for *C. auris*, *C. glabrata*, *C. guilliermondii*, *C. lusitaniae*, and *C. parapsilosis* (Lortholary et al., 2011; Pemán et al., 2012; Pfaller et al., 2012; Alexander et al., 2013; Pham et al., 2014; Dudiuk et al., 2017; Ruiz-Gaitán et al., 2019; Tortorano et al., 2021). MDR *Candida* isolates complicate clinical decision-making and are associated with treatment failure and high mortality rates (Lortholary et al., 2011; Alexander et al., 2013; Shields et al., 2013; Quindós et al., 2018; Tortorano et al., 2021).

Ibrexafungerp is a novel orally bioavailable semi-synthetic derivative of the terpenoid enfumafungin that inhibits glucan synthase, decreasing 1,3-β-D-glucan polymers and weakening fungal cell wall (Jiménez-Ortigosa et al., 2014; Pfaller et al., 2017; Schell et al., 2017; Davis et al., 2020). The current study that has evaluated European blood isolates confirms and extends the observations published in previous studies (Pfaller et al., 2013; Jiménez-Ortigosa et al., 2014; Berkow et al., 2017; Larkin et al., 2017; Marcos-Zambrano et al., 2017; Pfaller et al., 2017; Schell et al., 2017; Nunnally et al., 2019; Gamal et al., 2021). However, these previous studies have mostly tested American isolates. Our study with European isolates is in line with the conclusion that ibrexafungerp displays potent *in vitro* activity against the most clinically relevant species of *Candida*.

Ibrexafungerp showed an excellent activity against the *C. albicans* isolates included in the present study (MICs range 0.016–0.5 mg/L). These results confirm previous findings by other authors, such as Jiménez-Ortigosa et al. (2014); Schell et al. (2017), and Mesquida et al. (2021), demonstrating that ibrexafungerp showed activity against most *FKS*-mediated echinocandin-resistant *C. albicans* and against azole-resistant *C. albicans*.

Although there are no clinical breakpoints (CBPs) or ECVs available for ibrexafungerp, the study by Mesquida et al. (2022) as well as the present work have proposed WTULs. There are no differences in the proposed limits for *C. glabrata* (1 mg/L). However, our study suggests higher WTUL for *C. albicans* (0.5 mg/L vs. 0.25 mg/L by Mesquida et al.), for *C. parapsilosis* and *C. tropicalis* (2 mg/L vs. 1 mg/L by Mesquida et al.), and for *C. krusei* (4 mg/L vs. 2 mg/L by Mesquida et al.).

*C. parapsilosis* is the first or second etiology of IC in China, Japan, Latin America, and the Mediterranean countries of Africa, Asia, and Europe, such as Italy, Portugal, and Spain (Quindós et al., 2018). In the current report, ibrexafungerp MICs ranged from 0.016 mg/L to 8 mg/L. Our results are in accordance to those by Marcos-Zambrano et al. (2017). In both studies, ibrexafungerp displayed remarkably lower MICs than micafungin against *C. parapsilosis*. Although Mesquida et al. (2022) did not differentiate among *C. parapsilosis* species complex, they also reported lower MICs for ibrexafungerp than for echinocandins. These high MIC values reported for echinocandins against *C. parapsilosis* have been associated with substitutions in the hs1 region of *FKS1* (Garcia-Effron et al., 2008; Dudiuk et al., 2017; Marcos-Zambrano et al., 2017; Mesquida et al., 2022). Schell et al. (2017) also reported lower ibrexafungerp MIC_90_ values for 19 C*. parapsilosis* blood isolates (0.25 mg/L) compared with echinocandins, suggesting that changes in *FKS1* may not affect the capacity of ibrexafungerp to inhibit glucan synthase in this species.

*C. glabrata* has increased its etiological importance in IC in Australia, Canada, the USA, and countries in Central and Northern Europe, such as Belgium and Germany (Quindós, 2014; Trouvé et al., 2017; Quindós et al., 2018). In our study, ibrexafungerp displayed potent activity (MIC range 0.016–8 mg/L) also against fluconazole-resistant isolates. No cross-resistance has been found between ibrexafungerp and fluconazole, as previously noted by Marcos-Zambrano et al. (2017). The incidence of echinocandin resistance in *C. glabrata* is generally considered low, approximately 3%–4%, but can be as high as 30% in specific institutions (Alexander et al., 2013; Pham et al., 2014; Arendrup and Patterson, 2017). In the current study, ibrexafungerp MIC was ≤4 mg/L against four *C. glabrata* resistant to both fluconazole and micafungin. Pfaller et al. (2013) found similar results to ours but, in their report, ibrexafungerp was 8-fold more active than caspofungin against *C. glabrata*. Furthermore, ibrexafungerp showed activity against 31 C*. glabrata* strains with mutations in the hs of *FKS1* or *FKS2* (MIC ≤ 2 mg/L) and against 14 strains resistant to both caspofungin and fluconazole. In a later study by the same authors (Pfaller et al., 2017), 20 out of 25 *FKS* mutant *C. glabrata* isolates (80%) were NWT to one or more echinocandins, but only six (24%) were NWT to ibrexafungerp. Isolates of *C. glabrata* for which the ibrexafungerp MIC was > 2 mg/L (NWT) all were NWT and either intermediate or resistant to anidulafungin, caspofungin, and micafungin. In our study, 3 out of 4 isolates potentially NWT for ibrexafungerp were inhibited by ≤1 mg/L of caspofungin and one by 0.03 mg/L of micafungin. However, three of these four ibrexafungerp NWT isolates were resistant to micafungin.

Schell et al. (2017) evaluated 34 echinocandin-resistant *C. glabrata* isolates along with 34 paired control *C. glabrata* isolates, observing that ibrexafungerp MICs for individual *C. glabrata* isolates tended to be three to five dilutions higher than those for the echinocandins. However, ibrexafungerp MICs trended in agreement with those for the echinocandins. These authors detected that *C. glabrata* isolates with *FKS1* or *FKS2* mutations or echinocandin resistance were inhibited by ≤4 mg/L of ibrexafungerp. Nunnally et al. (2019) also reported good ibrexafungerp activity against 89 C*. glabrata* isolates with *FKS1* or *FKS2* mutations that conferred resistance to at least one echinocandin. Ibrexafungerp MIC values ranged from <0.03 mg/L to 4 mg/L while caspofungin and micafungin MICs ranged from 0.03 to >16 mg/L and 0.008 to >16 mg/L, respectively. In the study by Mesquida et al. (2022), an isolate of *C. glabrata* that displayed an ibrexafungerp MIC of 2 mg/L and echinocandin NWT phenotype harbored a mutation at *FKS2*. The spectrum of resistance mutations found in *C. glabrata* suggested a partially overlapping but independent binding site for ibrexafungerp relative to echinocandins on glucan synthase as these drugs are structurally dissimilar and interact differently with the target (Jiménez-Ortigosa et al., 2014). Consequently, this potent *in vitro* activity for ibrexafungerp has been reported against *C. glabrata* isolates harboring *FKS1* and *FKS2* point mutations that cause echinocandin resistance (Marcos-Zambrano et al., 2017; Pfaller et al., 2017; Schell et al., 2017; Nunnally et al., 2019; Mesquida et al., 2022). Moreover, the *in vitro* efficacy of ibrexafungerp has been supported by successful treatments in murine IC caused by *C. glabrata* resistant to echinocandins (Lepak et al., 2015; Wiederhold et al., 2018). In a murine model of IC caused by *C. albicans*, *C. parapsilosis*, or *C. glabrata* using an oral therapy with ibrexafungerp, Lepak et al. (2015) demonstrated that the AUC/MIC was the best pharmacodynamics parameter predicting clinical response. A MIC ≤ 1 mg/L obtained by CLSI would predict a clinical response using oral ibrexafungerp (Marcos-Zambrano et al., 2017).

In Asia, *C. tropicalis* was the second etiological agent of IC in many hospitals from China, India, Singapore, Thailand, and Taiwan (Quindós et al., 2018). We observed that ibrexafungerp MICs ranged from 0.06 mg/L to ≥ 8 mg/L for 40 C*. tropicalis* blood isolates, which confirms the consistently low ibrexafungerp MICs for azole-resistant *C. tropicalis* reported by Schell et al. (2017). Mesquida et al. (2022) found mutations in the *FKS1* gene of two NWT isolates of *C. tropicalis* that resulted, in both cases, in ibrexafungerp MICs between 0.5 and 1 mg/L.

We observed high activity of ibrexafungerp against 29 C*. krusei* (MIC range 0.125–1 mg/L). Schell et al. (2017) reported ibrexafungerp MICs for six isolates of *C. krusei* (MIC range from 0.5 mg/L to 4 mg/L), which were higher than those for the other *Candida* species. These authors suggested that a naturally occurring unidentified substitution may be responsible for the reduced activity of ibrexafungerp on glucan synthase in *C. krusei*. However, it is unknown if this translates into clinical failure.

In a recent Spanish nationwide study on candidemia, *C. orthopsilosis* was the fifth most frequently isolated species, preceding *C. krusei* (Pemán et al., 2012). In the present study, ibrexafungerp showed good activity against 20 C*. orthopsilosis* (MIC range 0.06–4 mg/L). To our knowledge, this is the first study on *in vitro* activity of ibrexafungerp against *C. orthopsilosis*. The lack of previous reports in this regard precludes comparison. Differences in ibrexafungerp activity against the closely related species *C. orthopsilosis* and *C. parapsilosis* highlights the importance of a correct identification of the *Candida* species involved in invasive infections.

*C. auris* is an emerging pathogen that has been identified in many countries associated with high mortality and a marked ability to develop resistance to multiple commonly used antifungal agents and to withstand standard infection control practices (Larkin et al., 2017; Ruiz-Gaitán et al., 2017; Ruiz-Gaitán et al., 2018; Ruiz-Gaitán et al., 2019). Data from Larkin et al. (2017) show that the *C. auris* isolates exhibited multidrug resistance against fluconazole and amphotericin B. Moreover, some isolates also exhibited high MIC values for voriconazole and itraconazole. In the present report, all *C. auris* isolates were resistant to fluconazole while ibrexafungerp showed notable activity (MIC range 0.5 mg/L to 8 mg/L). The *in vitro* activity of ibrexafungerp against European *C. auris* isolates from our study was similar to that described by Berkow et al. (2017) who studied 100 isolates of this species from Asia, Africa, and America. Of note, in the current study, MM values between *C. auris* WT and NWT isolates increased 64-fold for caspofungin and 4-fold for micafungin, whereas MM values for ibrexafungerp did not change. Interestingly, ibrexafungerp has been shown to be effective *in vivo* in the treatment of experimental infections with *C. auris* (Wiederhold et al., 2021).

Ibrexafungerp was twofold less active than caspofungin or micafungin against six *C. guilliermondii* blood isolates. Accordingly, Mesquida et al. (2022) also found higher ibrexafungerp MICs for 12 C*. guilliermondii* isolates, from 0.125 to 8 mg/L. Naturally occurring high echinocandins MICs against *C. guilliermondii* have been recognized since these antifungal agents were first introduced probably in association to substitutions in the hs1 region of *FKS1* (Dudiuk et al., 2017; Schell et al., 2017). In addition, Schell et al. (2017) reported that ibrexafungerp MIC ranges for three *C. lusitaniae* blood isolates were 1 to 2 mg/L and ibrexafungerp MICs for *C. lusitaniae* were three to five dilutions higher than those for the other species of *Candida*. Ibrexafungerp MICs for the two isolates of *C. lusitaniae* (MICs 4 and 8 mg/L) tested in the current study were higher than those observed for the other *Candida* species.

Aside from emerging resistance, an important limitation of echinocandins is that they must be administered daily by intravenous infusion, potentially prolonging hospital stays for patients undergoing echinocandin therapy and limiting them to inpatient settings in most instances. Of note, Wring et al. (2018) reported that the risk for interactions of ibrexafungerp with drugs metabolized *via* the cytochrome P450 family of enzymes is low. Ibrexafungerp exhibited concentration- and time-dependent fungicidal activity against *C. albicans*, *C. glabrata*, *C. krusei*, *C. parapsilosis*, and *C. tropicalis* in time-kill curve studies (Scorneaux et al., 2017). Moreover, ibrexafungerp has demonstrated efficacy for IC in phase 2 and 3 clinical studies (Spec et al., 2019).

The current study demonstrates that ibrexafungerp shows potent *in vitro* activity against *Candida* blood isolates and its activity is comparable to that of micafungin. Ibrexafungerp even exhibits good activity against fluconazole-resistant *Candida* isolates. Moreover, echinocandin-resistant isolates exhibit ibrexafungerp MICs consistent with those of echinocandin-susceptible isolates. However, direct comparisons of ibrexafungerp MICs with those of other antifungal drugs should be interpreted with caution, as different drugs may produce diverse ranges of MICs and yet have equivalent clinical efficacy because of their differences in bioavailability and in PK/PD properties. Although Pfaller et al. (2013) reported >90% essential agreement between both methods, CLSI and EUCAST, the comparison between our results and those of American authors should consider that the EUCAST method tends to yield higher MICs than CLSI, regardless of the studied species. Although the most clinically relevant species of *Candida* have been included in the current study, it would be interesting to assess the activity of ibrexafungerp against additional species as well as more NWT isolates.

In conclusion, we demonstrated that ibrexafungerp, a potent inhibitor of glucan synthase, could be an important acquisition to the antifungal toolbox for the therapy of patients suffering from IC caused by MDR species, such as *C. auris*, *C. glabrata*, or *C. krusei*. Considering the excellent pharmacokinetic properties of ibrexafungerp (oral availability and excellent tissue distribution and concentrations) as well as the potent activity observed against the main species causing candidiasis, ibrexafungerp should be regarded as a potential candidate for the therapy of these important diseases.

## Data Availability Statement

The raw data supporting the conclusions of this article will be made available by the authors, without undue reservation.

## Author Contributions

GQ, KM-C, and RS-M designed the research and participated in manuscript writing. KB-E, EC, MJL-S, AH, IM, AT, AP, MV-G, CM-A, AG, FS-R, JM-B, MR-I, EM-M, CC-M, LL-S, AR-G, MF-R, DL, JC, AR, JaP, JG, JoP, CP, OR, GE, NJ, DA, and EE conducted sampling and clinical measures, carried out the experiments, analyzed data, and drafted the manuscript. All authors contributed to the article and approved the submitted version.

## Funding

This study received funding from SCYNEXIS. The funder was not involved in the study design, collection, analysis, interpretation of data, the writing of the article, or the decision to submit it for publication. CM-A is a recipient of a grant from Fundación ONCE (Oportunidad al Talento). EE, AG, NJ, CM-A, and GQ have received grant support from Consejería de Educación, Universidades e Investigación del Gobierno Vasco (GIC15 IT-990-16), Fondo de Investigación Sanitaria del Gobierno de España (FIS PI11/00203), and UPV/EHU (UFI 11/25). All authors declare no other competing interests.

## Conflict of Interest

Outside the current study, we declare the following potential conflicts: GQ has received research grants from Astellas Pharma, Pfizer, Merck Sharp & Dohme, and SCYNEXIS. GQ has served on advisory/consultant boards for Merck, Sharp & Dohme, and SCYNEXIS, and he has received speaker honoraria from Abbvie, Astellas Pharma, Merck Sharp & Dohme, Pfizer, and SCYNEXIS. KB-E and DA are employed by SCYNEXIS.

The remaining authors declare that the research was conducted in the absence of any commercial or financial relationships that could be construed as a potential conflict of interest.

## Publisher’s Note

All claims expressed in this article are solely those of the authors and do not necessarily represent those of their affiliated organizations, or those of the publisher, the editors and the reviewers. Any product that may be evaluated in this article, or claim that may be made by its manufacturer, is not guaranteed or endorsed by the publisher.

## Abbreviations

ATCC, American Type Culture Collection; CBP, clinical breakpoints; DMSO, dimethyl sulfoxide; ECV, epidemiological cutoff values; EUCAST, European Committee on Antimicrobial Susceptibility Testing; GM, geometric mean; IC, invasive candidiasis; ITS, internal transcribed spacer; MALDI-TOF, matrix-assisted laser desorption/ionization–time-of-flight; MM, modal MIC; MIC, minimum inhibitory concentration; MOPS, morpholinopropane sulphate; NWT, non-wild type; PK/PD, pharmacokinetics and pharmacodynamics; QC, quality control; UPV/EHU, Universidad del País Vasco/Euskal Herriko Unibertsitatea; WT, wild type; WTUL, wild-type upper limits.

## References

[B1] Alexander B. D. Johnson M. D. Jiménez-Ortigosa C. Catania J. Booker R. Castanheira M.. (2013). Increasing Echinocandin Resistance in *Candida Glabrata*: Clinical Failure Correlates With Presence of *FKS* Mutations and Elevated Minimum Inhibitory Concentrations. Clin. Infect. Dis. 56, 1724–1732. doi: 10.1093/cid/cit136 23487382 PMC3658363

[B2] Arendrup M. C. Friberg N. Mares M. Kahlmeter G. Meletiadis J. Guinea J. (2020a). How to Interpret MICs of Antifungal Compounds According to the Revised Clinical Breakpoints V. 10.0 European Committee on Antimicrobial Susceptibility Testing (EUCAST). Clin. Microbiol. Infect. 26, 1464–1472. doi: 10.1016/j.cmi.2020.06.007 32562861

[B3] Arendrup M. C. Meletiadis J. Mouton J. W. Lagrou K. Hamal P. Guinea J. (2020b) EUCAST Definitive Document E.DEF 7.3.2 Method for the Determination of Broth Dilution Minimum Inhibitory Concentrations of Antifungal Agents for Yeasts. Available at: https://www.eucast.org/astoffungi/methodsinantifungalsusceptibilitytesting/susceptibility_testing_of_yeasts/ (Accessed 07 March 2022).

[B4] Arendrup M. C. Patterson T. F. (2017). Multidrug-Resistant *Candida*: Epidemiology, Molecular Mechanisms, and Treatment. J. Infect. Dis. 216 3), S445–S451. doi: 10.1093/infdis/jix131 28911043

[B5] Berkow E. L. Angulo D. Lockhart S. R. (2017). *In Vitro* Activity of a Novel Glucan Synthase Inhibitor, SCY-078, Against Clinical Isolates of *Candida Auris* . Antimicrob. Agents Chemother. 61, e00435-17. doi: 10.1128/AAC.00435-17 28483955 PMC5487607

[B6] Davis M. R. Donnelley M. A. Thompson G. R. (2020). Ibrexafungerp: A Novel Oral Glucan Synthase Inhibitor. Med. Mycol. 58, 579–592. doi: 10.1093/mmy/myz083 31342066

[B7] Dudiuk C. Macedo D. Leonardelli F. Theill L. Cabeza M. S. Gamarra S.. (2017). Molecular Confirmation of the Relationship Between *Candida Guilliermondii* Fks1p Naturally Occurring Amino Acid Substitutions and Its Intrinsic Reduced Echinocandin Susceptibility. Antimicrob. Agents Chemother. 61, e02644-16. doi: 10.1128/AAC.02644-16 28242659 PMC5404515

[B8] Espinel-Ingroff A. Arendrup M. C. Pfaller M. A. Bonfietti L. X. Bustamante B. Canton E.. (2013). Interlaboratory Variability of Caspofungin MICs for Candida Spp. Using CLSI and EUCAST Methods: Should the Clinical Laboratory Be Testing This Agent? Antimicrob. Agents Chemother. 57, 5836–5842. doi: 10.1128/AAC.01519-13 24018263 PMC3837874

[B9] EUCAST (2020) European Committee on Antimicrobial Susceptibility Testing Breakpoint. Antifungal Agents: Tables for Interpretation of MICs Version 10.0. Available at: https://www.eucast.org/fileadmin/src/media/PDFs/EUCAST_files/AFST/Clinical_breakpoints/AFST_BP_v10.0_200204_updatd_links_200924.pdf (Accessed March 2022).

[B10] EUCAST (2021) MIC and Zone Diameter Distributions and ECOFFs. Available at: https://mic.eucast.org/search/ (Accessed March 2022).

[B11] Fuller J. Dingle T. C. Bull A. Shokoples S. Laverdière M. Baxter M. R.. (2019). Species Distribution and Antifungal Susceptibility of Invasive *Candida* Isolates From Canadian Hospitals: Results of the CANWARD 2011-16 Study. J. Antimicrob. Chemother. 74 (Suppl.4), iv48–iv54. doi: 10.1093/jac/dkz287 31505645

[B12] Gamal A. Chu S. McCormick T. S. Borroto-Esoda K. Angulo D. (2021). And Ghannoum, M Ibrexafungerp, A Novel Oral Triterpenoid Antifungal in Development: Overview of Antifungal Activity Against *Candida Glabrata* . A Front. Cell Infect. Microbiol. 11. doi: 10.3389/fcimb.2021.642358 PMC8006402 33791244

[B13] Garcia-Effron G. Katiyar S. K. Park S. Edlind T. D. Perlin D. S. (2008). A Naturally Occurring Proline-to-Alanine Amino Acid Change in Fks1p in *Candida Parapsilosis*, *Candida Orthopsilosis*, and *Candida Metapsilosis* Accounts for Reduced Echinocandin Susceptibility. Antimicrob. Agents Chemother. 52, 2305–2312. doi: 10.1128/AAC.00262-08 18443110 PMC2443908

[B14] Gil-Alonso S. Jauregizar N. Cantón E. Eraso E. Quindós G. (2015a). Comparison of the *In Vitro* Activity of Echinocandins Against *Candida Albicans*, *Candida Dubliniensis*, and *Candida Africana* by Time-Kill Curves. Diagn. Microbiol. Infect. Dis. 82, 57–61. doi: 10.1016/j.diagmicrobio.2015.01.010 25703894

[B15] Gil-Alonso S. Jauregizar N. Cantón E. Eraso E. Quindós G. (2015b). *In Vitro* Fungicidal Activities of Anidulafungin, Caspofungin, and Micafungin Against *Candida Glabrata*, *Candida Bracarensis*, and *Candida Nivariensis* Evaluated by Time-Kill Studies. Antimicrob. Agents Chemother. 59, 3615–3618. doi: 10.1128/AAC.04474-14 25801575 PMC4432200

[B16] Jiménez-Ortigosa C. Paderu P. Motyl M. R. Perlin D. S. (2014). Enfumafungin Derivative MK-3118 Shows Increased *In Vitro* Potency Against Clinical Echinocandin-Resistant *Candida* Species and *Aspergillus* Species Isolates. Antimicrob. Agents Chemother. 58, 1248–1251. doi: 10.1128/AAC.02145-13 24323472 PMC3910825

[B17] Jiménez-Ortigosa C. Perez W. B. Angulo D. Borroto-Esoda K. Perlin D. S. (2017). *De Novo* Acquisition of Resistance to SCY-078 in *Candida Glabrata* Involves *FKS* Mutations That Both Overlap and Are Distinct From Those Conferring Echinocandin Resistance. Antimicrob. Agents Chemother. 61, e00833–e00817. doi: 10.1128/AAC.00833-17 28630180 PMC5571364

[B18] Larkin E. Hager C. Chandra J. Mukherjee P. K. Retuerto M. Salem I.. (2017). The Emerging Pathogen *Candida Auris*: Growth Phenotype, Virulence Factors, Activity of Antifungals, and Effect of SCY-078, A Novel Glucan Synthesis Inhibitor, on Growth Morphology and Biofilm Formation. Antimicrob. Agents Chemother. 61, e02396-16. doi: 10.1128/AAC.02396-16 28223375 PMC5404565

[B19] Lepak A. J. Marchillo K. Andes D. R. (2015). Pharmacodynamic Target Evaluation of a Novel Oral Glucan Synthase Inhibitor, SCY-078 (MK-3118), Using an *In Vivo* Murine Invasive Candidiasis Model. Antimicrob. Agents Chemother. 59, 1265–1272. doi: 10.1128/AAC.04445-14 25512406 PMC4335824

[B20] Lortholary O. Desnos-Ollivier M. Sitbon K. Fontanet A. Bretagne S. Dromer F.. (2011). Recent Exposure to Caspofungin or Fluconazole Influences the Epidemiology of Candidemia: A Prospective Multicenter Study Involving 2,441 Patients. Antimicrob. Agents Chemother. 55, 532–538. doi: 10.1128/AAC.01128-10 21078946 PMC3028765

[B21] Marcos-Zambrano L. J. Gómez-Perosanz M. Escribano P. Bouza E. Guinea J. (2017). The Novel Oral Glucan Synthase Inhibitor SCY-078 Shows *In Vitro* Activity Against Sessile and Planktonic *Candida* Spp. J. Antimicrob. Chemother. 72, 1969–1976. doi: 10.1093/jac/dkx010 28175309

[B22] Mesquida A. Díaz-García J. Sánchez-Carrillo C. Muñoz P. Escribano P. Guinea J. (2022). *In Vitro* Activity of Ibrexafungerp Against *Candida* Species Isolated From Blood Cultures. Determination of Wild-Type Populations Using the EUCAST Method. Clin. Microbiol. Infect. 28, 140.e1–140.e4. doi: 10.1016/j.cmi.2021.09.030 34619396

[B23] Mesquida A. Vicente T. Reigadas E. Palomo M. Sánchez-Carrillo C. Muñoz P.. (2021). *In Vitro* Activity of Ibrexafungerp and Comparators Against *Candida Albicans* Genotypes From Vaginal Samples and Blood Cultures. Clin. Microbiol. Infect. 27, 915.e5–915.e8. doi: 10.1016/j.cmi.2021.02.006 33601007

[B24] Miranda-Zapico I. Eraso E. Hernández-Almaraz J. L. López-Soria L. M. Carrillo-Muñoz A. J. Hernández-Molina J. M.. (2011). Prevalence and Antifungal Susceptibility Patterns of New Cryptic Species Inside the Species Complexes *Candida Parapsilosis* and *Candida Glabrata* Among Blood Isolates From a Spanish Tertiary Hospital. J. Antimicrob. Chemother. 66, 2315–2322. doi: 10.1093/jac/dkr298 21795259

[B25] Nunnally N. S. Etienne K. A. Angulo D. Lockhart S. R. Berkow E. L. (2019). *In Vitro* Activity of Ibrexafungerp, A Novel Glucan Synthase Inhibitor Against *Candida Glabrata* Isolates With *FKS* Mutations. Antimicrob. Agents Chemother. 63, e01692-19. doi: 10.1128/AAC.01692-19 PMC6811432 31481447

[B26] Pemán J. Cantón E. Quindós G. Eraso E. Alcoba J. Guinea J.. (2012). Epidemiology, Species Distribution, and *In Vitro* Antifungal Susceptibility of Fungaemia in a Spanish Multicentre Prospective Survey. J. Antimicrob. Chemother. 67, 1181–1187. doi: 10.1093/jac/dks019 22351683

[B27] Pfaller M. A. Castanheira M. Lockhart S. R. Ahlquist A. M. Messer S. A. Jones R. N. (2012). Frequency of Decreased Susceptibility and Resistance to Echinocandins Among Fluconazole-Resistant Bloodstream Isolates of *Candida Glabrata* . J. Clin. Microbiol. 50, 1199–1203. doi: 10.1128/JCM.06112-11.O 22278842 PMC3318516

[B28] Pfaller M. A. Diekema D. J. Turnidge J. D. Castanheira M. Jones R. N. (2019). Twenty Years of the SENTRY Antifungal Surveillance Program: Results for *Candida* Species From 1997-2016. Open Forum Infect. Dis. 6 1), S79–S94. doi: 10.1093/ofid/ofy358 30895218 PMC6419901

[B29] Pfaller M. A. Messer S. A. Motyl M. R. Jones R. N. Castanheira M. (2013). Activity of MK-3118, A New Oral Glucan Synthase Inhibitor, Tested Against *Candida* Spp. By Two International Methods (CLSI and EUCAST). J. Antimicrob. Chemother. 68, 858–863. doi: 10.1093/jac/dks466 23190764

[B30] Pfaller M. A. Messer S. A. Rhomberg P. R. Borroto-Esoda K. Castanheira M. (2017). Differential Activity of the Oral Glucan Synthase Inhibitor SCY-078 Against Wild-Type and Echinocandin-Resistant Strains of *Candida* Species. Antimicrob. Agents Chemother. 61, e00161-17. doi: 10.1128/AAC.00161-17 28533234 PMC5527608

[B31] Pham C. D. Iqbal N. Bolden C. B. Kuykendall R. J. Harrison L. H. Farley M. M.. (2014). The Role of *FKS* Mutations in *C. Glabrata*: MIC Values, Echinocandin Resistance and Multidrug Resistance. Antimicrob. Agents Chemother. 58, 4690–4696. doi: 10.1128/AAC.03255-15 24890592 PMC4136002

[B32] Quindós G. (2014). Epidemiology of Candidaemia and Invasive Candidiasis. A Changing Face. Rev. Iberoam. Micol. 31, 42–48. doi: 10.1016/j.riam.2013.10.001 24270071

[B33] Quindós G. Marcos-Arias C. San-Millán R. Mateo E. Eraso E. (2018). The Continuous Changes in the Aetiology and Epidemiology of Invasive Candidiasis: From Familiar *Candida Albicans* to Multiresistant *Candida Auris* . Int. Microbiol. 21, 107–119. doi: 10.1007/s10123-018-0014-1 30810955

[B34] Ruiz-Gaitán A. C. Cantón E. Fernández-Rivero M. E. Ramírez P. Pemán J. (2019). Outbreak of *Candida Aur*is in Spain: A Comparison of Antifungal Activity by Three Methods With Published Data. Int. J. Antimicrob. Agents 53, 541–546. doi: 10.1016/j.ijantimicag.2019.02.005 30769198

[B35] Ruiz-Gaitán A. C. Moret A. López Hontangas J. L. Molina J. M. Aleixandre López A. I. Cabezas A. H.. (2017). Nosocomial Fungemia by *Candida Auris*: First Four Reported Cases in Continental Europe. Rev. Iberoam. Micol. 34, 23–27. doi: 10.1016/j.riam.2016.11.002 28131716

[B36] Ruiz-Gaitán A. Moret A. M. Tasias-Pitarch M. Aleixandre-López A. I. Martínez-Morel H. Calabuig E.. (2018). An Outbreak Due to *Candida Auris* With Prolonged Colonisation and Candidaemia in a Tertiary Care European Hospital. Mycoses 61, 498–505. doi: 10.1111/myc.12781 29655180

[B37] Schell W. A. Jones A. M. Borroto-Esoda K. Alexander B. D. (2017). Antifungal Activity of SCY-078 and Standard Antifungal Agents Against 178 Clinical Isolates of Resistant and Susceptible *Candida* Species. Antimicrob. Agents Chemother. 61, e01102-17. doi: 10.1128/AAC.01102-17 28827419 PMC5655100

[B38] Schwebke J. R. Sobel R. Gersten J. K. Sussman S. A. Lederman S. N. Jacobs M. A.. (2021). Ibrexafungerp Versus Placebo for Vulvovaginal Candidiasis Treatment: A Phase 3, Randomized, Controlled Superiority Trial (VANISH 303). Clin. Infect. Dis. Ciab. 750. doi: 10.1093/cid/ciab750 PMC9187327 34467969

[B39] Scorneaux B. Angulo D. Borroto-Esoda K. Ghannoum M. Peel M. Wring S. (2017). SCY-078 Is Fungicidal Against *Candida* Species in Time-Kill Studies. Antimicrob. Agents Chemother. 61, e01961-16. doi: 10.1128/AAC.01961-16 28069658 PMC5328566

[B40] Shields R. K. Nguyen M. H. Press E. G. Updike C. L. Clancy C. J. (2013). Caspofungin MICs Correlate With Treatment Outcomes Among Patients With *Candida Glabrata* Invasive Candidiasis and Prior Echinocandin Exposure. Antimicrob. Agents Chemother. 57, 3528–3535. doi: 10.1128/AAC.00136-13 23669387 PMC3719767

[B41] Sobel R. Nyirjesy P. Ghannoum M. A. Delchev D. A. Azie N. E. Angulo D.. (2022). Efficacy and Safety of Oral Ibrexafungerp for the Treatment of Acute Vulvovaginal Candidiasis: A Global Phase 3, Randomised, Placebo-Controlled Superiority Study (VANISH 306). BJOG 129, 412–420. doi: 10.1111/1471-0528.16972 34676663 PMC9299454

[B42] Spec A. Pullman J. Thompson G. R. Powderly W. G. Tobin E. H. Vazquez J.. (2019). MSG-10: A Phase 2 Study of Oral Ibrexafungerp (SCY-078) Following Initial Echinocandin Therapy in Non-Neutropenic Patients With Invasive Candidiasis. J. Antimicrob. Chemother. 74, 3056–3062. doi: 10.1093/jac/dkz277 31304536

[B43] Tortorano A. M. Prigitano A. Morroni G. Brescini L. Barchiesi F. (2021). Candidemia: Evolution of Drug Resistance and Novel Therapeutic Approaches. Infect. Drug Resist. 14, 5543–5553. doi: 10.2147/IDR.S274872 34984009 PMC8702982

[B44] Trouvé C. Blot S. Hayette M. P. Jonckheere S. Patteet S. Rodriguez-Villalobos H.. (2017). Epidemiology and Reporting of Candidaemia in Belgium: A Multi-Centre Study. Eur. J. Clin. Microbiol. Infect. Dis. 36, 649–655. doi: 10.1007/s10096-016-2841-3 27858242

[B45] Wiederhold N. P. (2021). Antifungal Susceptibility Testing: A Primer for Clinicians. Open Forum Infect. Dis. 8, ofab444. doi: 10.1093/ofid/ofab444 34778489 PMC8579947

[B46] Wiederhold N. P. Najvar L. K. Jaramillo R. Olivo M. Pizzini J. Catano G.. (2018). Oral Glucan Synthase Inhibitor SCY-078 Is Effective in an Experimental Murine Model of Invasive Candidiasis Caused by WT and Echinocandin-Resistant *Candida Glabrata* . J. Antimicrob. Chemother. 73, 448–451. doi: 10.1093/jac/dkx422 29177447 PMC5890676

[B47] Wiederhold N. P. Najvar L. K. Olivo M. Morris K. N. Patterson H. P. Catano G.. (2021). Ibrexafungerp Demonstrates *In Vitro* Activity Against Fluconazole-Resistant *Candida Auris* and *In Vivo* Efficacy With Delayed Initiation of Therapy in an Experimental Model of Invasive Candidiasis. Antimicrob. Agents Chemother. 65, e02694-20. doi: 10.1128/AAC.02694-20 33753333 PMC8315906

[B48] Wring S. Murphy G. Atiee G. Corr C. Hyman M. Willett M.. (2018). Lack of Impact by SCY-078, a First-in-Class Oral Fungicidal Glucan Synthase Inhibitor, on the Pharmacokinetics of Rosiglitazone, A Substrate for CYP450 2C8, Supports the Low Risk for Clinically Relevant Metabolic Drug-Drug Interactions. J. Clin. Pharmacol. 58, 1305–1313. doi: 10.1002/jcph.1146 29746713 PMC6175093

