# Supplementary material for: In Vitro Antifungal Activity of Ibrexafungerp (SCY-078) Against Contemporary Blood Isolates From Medically Relevant Species of Candida: A European Study
Source: Front Cell Infect Microbiol. 2022 May 16;12:906563. doi: 10.3389/fcimb.2022.906563 (PMC9149255; doi:10.3389/fcimb.2022.906563)
Supplement: Supplementary file 1 [file Table_1.docx]

**Table S1.** Ibrexafungerp MIC distributions for the quality control strains.

| **Antifungal drug** | **No. of occurrences at MIC (mg/L) Ibrexafungerp** | | | | | | | | | | | | **MIC** | |
| --- | --- | --- | --- | --- | --- | --- | --- | --- | --- | --- | --- | --- | --- | --- |
|  | **≤0.008** | **0.016** | **0.03** | **0.06** | **0.125** | **0.25** | **0.5** | **1** | **2** | **4** | **8** | **16** | **50%** | **90%** |
|  | | | | | | | | | | | | | | |
| *Candida parapsilosis* ATCC 22019 | 0 | 0 | 0 | 0 | 1 | 2 | 6 | 11 | 0 | 0 | 0 | 0 | 1 | 1 |
|  | | | | | | | | | | | | | | |
| *Candida krusei* ATCC 6258 | 0 | 0 | 0 | 0 | 0 | 1 | 3 | 15 | 0 | 0 | 0 | 0 | 1 | 1 |
|  | | | | | | | | | | | | | | |
| *Candida albicans* ATCC 64548 | 0 | 0 | 8 | 5 | 1 | 0 | 0 | 0 | 0 | 0 | 0 | 0 | 0.03 | 0.06 |
|  | | | | | | | | | | | | | | |
| *Candida albicans* ATCC 64550 | 0 | 0 | 12 | 1 | 0 | 0 | 0 | 0 | 0 | 0 | 0 | 0 | 0.03 | 0.03 |
